# Population Genomic Analysis Reveals a Highly Conserved Mitochondrial Genome in *Fusarium asiaticum*

**DOI:** 10.3389/fmicb.2020.00839

**Published:** 2020-05-05

**Authors:** Meixin Yang, Hao Zhang, Theo A. J. van der Lee, Cees Waalwijk, Anne D. van Diepeningen, Jie Feng, Balázs Brankovics, Wanquan Chen

**Affiliations:** ^1^State Key Laboratory for Biology of Plant Diseases and Insect Pests, Institute of Plant Protection, Chinese Academy of Agriculture Sciences, Beijing, China; ^2^Biointeractions and Plant Health, Wageningen Plant Research, Wageningen, Netherlands

**Keywords:** *Fusarium asiaticum*, mitogenome, introns, migrations, population genomics

## Abstract

*Fusarium asiaticum* is one of the pivotal members of the *Fusarium graminearum* species complex (FGSC) causing Fusarium head blight (FHB) on wheat, barley and rice in large parts of Asia. Besides resulting in yield losses, FHB also causes the accumulation of mycotoxins such as nivalenol (NIV) and deoxynivalenol (DON). The aim of this study was to conduct population studies on *F. asiaticum* from Southern China through mitochondrial genome analyses. All strains were isolated from wheat or rice from several geographic areas in seven provinces in Southern China. Based on geographic location and host, 210 isolates were selected for next generation sequencing, and their mitogenomes were assembled by GRAbB and annotated to explore the mitochondrial genome variability of *F. asiaticum*. The *F. asiaticum* mitogenome proves extremely conserved and variation is mainly caused by absence/presence of introns harboring homing endonuclease genes. These variations could be utilized to develop molecular markers for track and trace of migrations within and between populations. This study illustrates how mitochondrial introns can be used as markers for population genetic analysis. SNP analysis demonstrate the occurrence of mitochondrial recombination in *F. asiaticum* as was previously found for *F. oxysporum* and implied for *F. graminearum*. Furthermore, varying degrees of genetic diversity and recombination showed a high association with different geographic regions as well as with cropping systems. The mitogenome of *F. graminearum* showed a much higher SNP diversity while the interspecies intron variation showed no evidence of gene flow between the two closely related and sexual compatible species.

## Introduction

Members of the *Fusarium graminearum* species complex (FGSC) are the main causal agents of *Fusarium* head blight (FHB) and infect wheat (*Triticum aestivum* L.), maize (*Zea mays* L.) and other small grain cereals, causing significant losses in grain quality and yield around the world (Goswami and Kistler, 2004). In addition, infected crops often accumulate mycotoxins, such as deoxynivalenol, zearalenone and nivalenol, which pose a serious health risk to animals and humans (D’Mello et al., 1999). FGSC comprises at least 16 distinct species (Sarver et al., 2011). The most important member is the globally occurring species *F. graminearum*. The second most important species is *F. asiaticum*, the main causal agent of FHB in Asia including China, Korea, Nepal, and Japan (van der Lee et al., 2015). *F. asiaticum* is particularly dominant in the Middle-Lower Reaches of the Yangtze River (MLRYR) and southwest of China, where FHB is the most prevalent (Zhang et al., 2012). In a previous study, we have found that the specific crop rotations correlate with the occurrence of FGSC species, where wheat/rice rotations were found to be highly conducive for *F. asiaticum* (Zhang et al., 2016a).

Crop debris (wheat and rice straw, corn stalks, and debris from other crops) is a crucial element for *Fusarium* to complete its life cycle, since infested crop residues form the overwintering niches for FHB pathogens. In the next cropping season ascospores, the primary inoculum of FHB, are released from perithecia forming on the surface of crop debris (Schmale and Bergstrom, 2003). In Brazil and United States, *F. graminearum* was determined as the predominant species on wheat (Gale et al., 2011; Del Ponte et al., 2015), whereas *F. asiaticum* was exclusively associated with rice growing areas (Kuhnem et al., 2016). In southern China where rice is the predominant crop grown, *F. asiaticum* is widespread, while *F. graminearum* is mainly found in Northern China, where wheat and maize are cultivated in monoculture or in rotation (Zhang et al., 2012).

Several population studies have shown shifts and high dynamics in *Fusarium* populations (Waalwijk et al., 2003; Gale et al., 2007; Ward et al., 2008). In Canada, a rapid displacement of 15ADON producing *F. graminearum* by a newly introduced 3ADON-producing *F. graminearum* population was observed (Ward et al., 2008). While in the South of the United States an influx of NIV producers, both *F. asiaticum* and *F. graminearum*, was reported (Gale et al., 2011). In our previous studies, NIV-producing *F. asiaticum* were shown to be replaced by 3ADON-producing *F. asiaticum* from east to west in China (Zhang et al., 2012). Besides, changes in chemotype (or mycotoxin profile), fungicide resistance (Talas and McDonald, 2015; Zhang et al., 2016b) and virulence might be distinct between populations. Hence, it is important to monitor shift of different *Fusarium* populations (Gale et al., 2007; Zhang et al., 2012). This will aid the understanding of population dynamics and the evolutionary principles underlying the FHB outbreaks.

The “second genome” of eukaryotes, the mitochondrial genome, is a highly informative phylogenetic marker generally used for inferring evolutionary and phylogenetic relationships at diverse taxonomic levels (Basse, 2010). The high copy numbers of mitochondrial genomes (mitogenomes) within individual cells facilitate accessing their genomes (Gillett et al., 2014). Besides, homologous regions are easy to identify, as the mitogenome has a relatively simple composition (Avise et al., 1983). Mobile mitochondrial introns prove common in many fungi (Haugen et al., 2005) and though they may interfere with polymerase chain reaction amplification methods, in next-generation sequencing (NGS) they provide additional markers.

Significant differences exist in fungal mitogenomes related to genome structure, gene arrangement, repeat content and size (Li et al., 2018a, b, 2019). In previous studies of the genus *Fusarium*, mitogenomes show many common features: in most *Fusarium* spp., including *F. verticillioides*, *F. oxysporum*, *F. graminearum* and *F. solani*, mitogenomes harbor 14 protein coding genes, a wide range of tRNA coding genes and two rRNA coding genes, rns (mtSSU) and rnl (mtLSU). All of these genes are encoded on the same strand and genes are organized in the same order (Pantou et al., 2008; Al-Reedy et al., 2012). In our previous study of *Fusarium* mitogenomes, we have found that intraspecific differences in mitogenomes sizes are mainly due to the absence/presence of introns. Mitochondrial recombination has been described in *F. oxysporum* and *F. graminearum*. The spread of introns can take place by three different mechanisms: horizontal transfer, vertical inheritance and recombination (Al-Reedy et al., 2012; Brankovics et al., 2018). Therefore, mitogenomes of *Fusarium* are potential markers to track and trace the spread of populations.

To date, the mitogenome of *F. asiaticum* has not been assembled and hence the species’ variation and diversity of this mitogenome is unknown. In this study, we sequenced, *de novo* assembled and annotated mitogenomes of 210 *F. asiaticum* isolates collected from four ecological regions of China. Subsequently, variation analysis was conducted and a pan-mitogenome of *F. asiaticum* was created. The efficacy of using mitochondrial introns as markers for population genetic analysis was investigated, and combined with other mitochondrial variations the population diversity of *F. asiaticum* in Southern China was assessed. Finally, interspecies comparative mitochondrial genomic analyses were performed on 24 previously published mitogenomes of *F. graminearum* and the 210 mitogenomes of *F. asiaticum* generated in this study.

## Materials and Methods

### Isolates

Two hundred and ten *F. asiaticum* strains and twenty-four *F. graminearum* strains were analyzed in this study (Supplementary Table S1). The *F. asiaticum* strains were collected from six provinces in Southern China, which can be divided into four ecological regions: Hubei, Jiangsu, and Anhui Province are located in the Middle-Lower reaches of the Yangtze River (MLRYR) and regarded as one ecological region. The Hunan Province is a transition region between plains and mountains, its northern part is adjacent to MLRYR. The Sichuan and Fujian Provinces are both mountainous regions. The cropping system is also different among these regions. In the MLRYR, which is the main wheat producing area in Southern China, wheat-rice rotation is predominant. In Sichuan Province, crop diversity is much higher and the acreage of wheat is significantly smaller compared to MLRYR (Zeng et al., 2011). In Sichuan Province, wheat is rotated with rice, maize, soybean and several other crops, but wheat-rice rotation is the most common. In the MLRYR and Sichuan, *Fusarium* strains were isolated from diseased wheat heads and perithecia on rice stubble (debris of the previous crop) by a single-spore isolation procedure described previously (Yang et al., 2018). In Fujian and Hunan Provinces no wheat is grown, two harvests of rice/year is the prevalent cropping system and consequently all *Fusarium* strains isolated from this regions are from rice stubble. A detailed sampling information of these strains are shown in Supplementary Table S1.

### Sequencing

Whole genome sequencing of *F. asiaticum* isolates were performed by Biomarker Technologies Corporation (Beijing, China). Random sheared shotgun library with specific index of each *F. asiaticum* strain was made using the Illumina TruSeq DNA Sample Prep Kit, according to manufacturer’s protocols (Illumina). The concentration of independent genomic library was quantitated by QUBIT 3.0 fluorometer. The 210 independent libraries were pooled with equal amount by adjusting the solution volume and then were sequenced using the Illumina HiSeq X Ten platform which supported the acquisition of 2 × 150 bp paired-end reads at ∼50X coverage per sample. Subsequently, Casava 1.8 software was used to de-multiplex reads which were filtered for quality using *fastp* with forced polyG tail trimming (*-g*) and minimum quality Phred score ≥ 20 (*-q 20*). The *F. graminearum* strains were sequenced by other research groups, and their mitochondrial genome sequences were downloaded and analyzed in this study (Yuen and Mila, 2015; Wang et al., 2017; Brankovics et al., 2018).

### Mitochondrial Genome Assembly

The mitogenomes of the sequenced strains were assembled by GRAbB (Genomic Region Assembly by Baiting) with the SPAdes assembler (Bankevich et al., 2012; Nurk et al., 2013; Brankovics et al., 2016), which had also been used for mitogenome assembly of *F. graminearum* strains (Brankovics et al., 2018). Isolate PH-1 (HG970331) was used as the reference sequence to bait the mitogenome sequences. The assembled contigs were joined based on overlap to form a single contig using the helper scripts from the GRAbB package^1^. The final overlapping sequence was clipped during the process, which confirmed that all sequences were derived from a circular configuration.

### Mitochondrial Genome Annotation

Mitochondrial genome annotation was performed as described by Brankovics et al., 2018. Briefly, MFannot^2^, NCBI’s ORF Finder^3^, CD-Search (Marchler-Bauer and Bryant, 2004), InterPro (Mitchell et al., 2015), tRNAscan-SE (Pavesi et al., 1994) were combined to annotate the mitogenome sequence. The annotation information of the mitogenomes has been deposited at GenBank: MN935220-MN935429. GenBank accession numbers are also listed in Supplementary Table S1.

### Variation Analysis of Mitogenomes

The mitogenomes of *F. asiaticum* and *F. graminearum* strains were aligned using MUSCL v3.8.31 (Edgar, 2004) and manually curated. The alignment was used to extract variations using the *msa2vcf* function of Jvarkit^4^. Subsequently, variant locations in exonic, intronic, or intergenic regions were identified. In the case of exonic variants, they were characterized as synonymous or non-synonymous variation and amino acid changes were noted. The structure and variant annotation were visualized using the *ggplot2* package in *R*.

Since intronic sequences behave as mobile genetic elements in fungal mitochondrial genomes we partitioned the analysis into two: one focusing on the intron sequences and the other on the non-intronic regions (intergenic and coding sequences).

### Intron Analysis

The intron size information for each strain was extracted from the annotation file and used as markers to analyze genotypic differentiation of introns of strains among different populations (Supplementary Table S3). Multilocus genotype (MLG), rarefaction Multilocus genotype (eMLG), allele frequencies, and Nei genetic distance analyses of the population from different ecological regions were conducted with GENALEX version 6.5 (Peakall and Smouse, 2012). Simpson’s index (Lambda), Nei’s gene diversity index (Hexp), value of the standardized index of association for each population factor (rbarD), *P*-value for rbarD from the number of reshuffles indicated in sample (p.rD), and principal component analysis (PCA) were performed with *R* package *Poppr* (Kamvar et al., 2014). The correlation analysis between geographical distance and genetic distance of *F. asiaticum* from 50 sampling sites was conducted by Mantel test in *R* package *ade4* (Mantel, 1967).

### Analysis of Non-intronic Regions

Intronic regions were clipped out from the mitogenome alignment and parsimony informative sites were extracted excluding gapped sites. The sites were checked manually, especially the SNP rich regions. A region where most of the SNPs could be traced to some strains containing longer poly tracts than other isolates was identified as poor quality region and removed from the downstream analysis. This region is also the common breakpoint in mitochondrial genome assemblies before circularization. Subsequently, the curated data was used for network construction using PopART (Population Analysis with Reticulate Trees) (Leigh et al., 2015). The geographic origin and the host information was used to color the network nodes.

### Phylogenetic Analysis

A *F. culmorum* mitogenome (accession number NC_026993.1) was added to the non-intronic alignment of the *F. asiaticum* and *F. graminearum* strains. The alignment was generated by MUSCLE v3.8.31. Phylogenetic analysis was conducted using the ML method which was run using IQ-Tree with model selection and 1000 bootstraps with the *F. culmorum* strain as outgroup. The best model selected was TPM2u + I + G4.

## Results

### Mitogenomes of *F. asiaticum*

#### Features of the *F. asiaticum* Mitogenome

The complete mitogenomes of all 210 *F. asiaticum* strains were assembled into single circular DNA molecules, with the total size ranging from 89,966 to 98,925 bp. All mitogenomes encoded the same set of genes: 14 Protein coding genes (PCGs), 28 tRNA, two rRNA, and one large open reading frame with unknown function (LV-uORF), all with the same orientation and order. The intronic regions vary in length from 44, 676 to 53,583 bp, which accounts for almost half of the entire length of the mitogenome in each strain.

#### Variations Among *F. asiaticum* Mitogenomes

**FIGURE 1:**
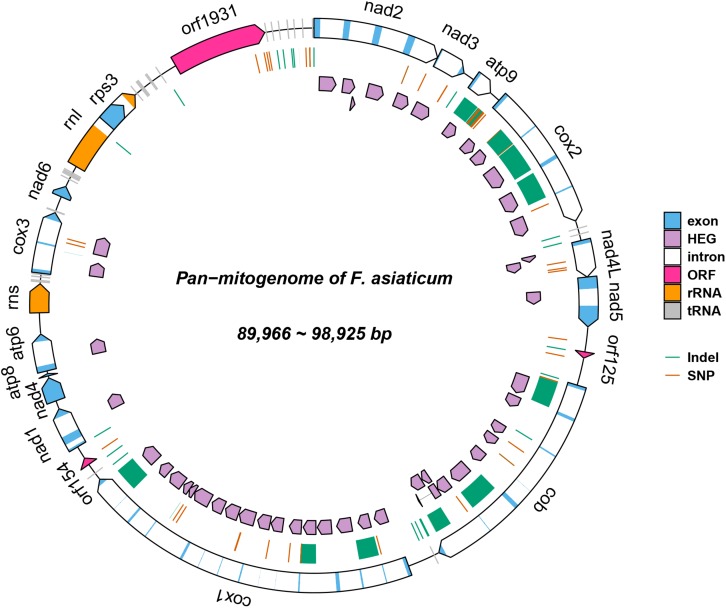
Pan-mitogenome of 210 *Fusarium asiaticum* isolates. This figure summarizes all the genetic variability observed within the species. Regions affected by indels are highlighted in green and SNPs are indicated in red.

A total of 120 polymorphic sites were identified in the 210 *F. asiaticum* mitogenomes, including 76 SNPs and 44 indels (Figure 1). There were 40 SNPs detected in intergenic regions and 22 SNPs were located in introns. Only 11 SNPs were identified in the conserved protein coding regions, showing a low level of variation and all of them were synonymous mutations. The remaining 3 SNPs were identified in the large variable open reading frame with unknown function (LVuORF or orf1931 region, among which two caused non-synonymous substitutions: at position 1648 from proline (CCT) to serine (TCT) and at position 1840 from arginine (CGC) to leucine (CTC) residues in the predicted protein. Indels were only detected in intergenic and intronic regions with sizes varying from 1 to 1994 bp. The vast majority of indels in intergenic regions were short; 24/26 were shorter than 4 bp, one was 16 bp and the largest 163 bp long. In sharp contrast, all large indels (>900 bp) were found in intronic regions totaling 14704 bp in length.

**FIGURE 2:**
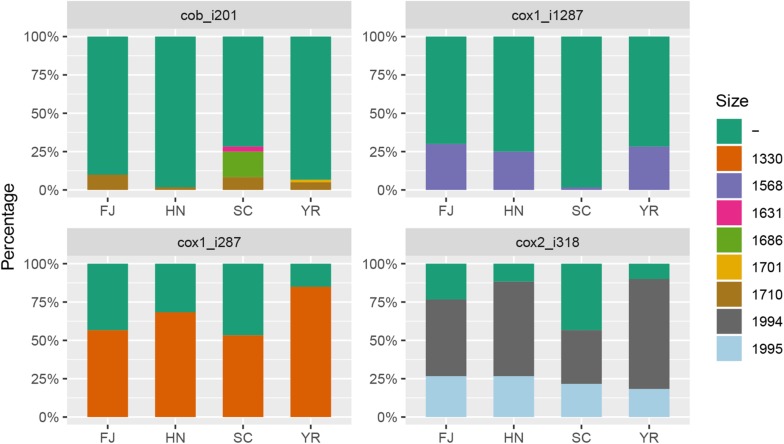
The size composition of four introns: cox2_i318, cob_i201, cox1_i287, cox1_i1287. FJ, Fujian; HN, Hunan; SC, Sichuan; YR, Middle-Lower Reaches of Yangtze River. -: absence, and numbers indicate length of introns in base pairs.

Within the 210 *F. asiaticum* mitogenomes a total of 38 introns were identified, 31 of them were present in all genomes while seven were variable. The number of introns in each individual isolate ranged from 31 to 36. Thirty-six strains contained the minimum number of 31 introns while only one strain contained the maximum number of 36 introns. The length variation of mitochondrial genome in *F. asiaticum* is mainly caused by variation in the intron regions. Three large indels (cox2-i228, cob-i823, and cox1-i621) are putatively due to an additional intron insertion inside an already present intron, because the insert region coded for an additional HEG. Among the seven introns which showed presence/absence pattern, three were absent in most isolates: two (atp9-i181 and cob-i779) were just present in one strain and one (cox2-i552) was found in four strains. We investigated the size composition of the other four introns. As shown in Figure 2, Sichuan showed a significantly higher ratio of intron absence than the other three regions for cox1-i287, cox1-i1287, and cox2-i318. While for cob-i201, Sichuan had the highest presence ratio and it was the only province with multiple haplotypes.

#### Analysis of Non-intronic Regions of the Mitogenomes of *F. asiaticum*

**FIGURE 3:**
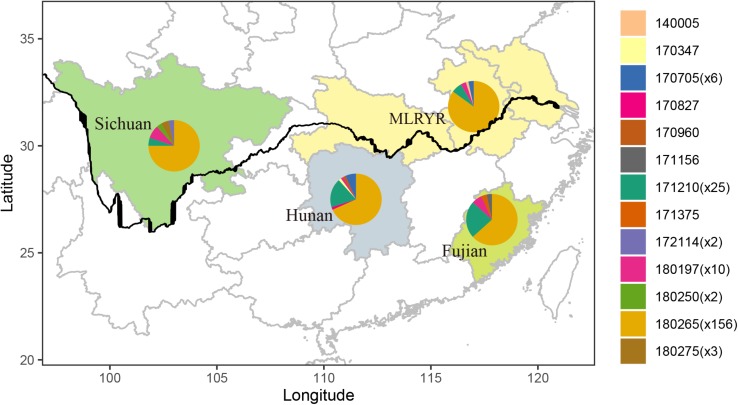
Map of four ecological regions indicating the distribution of haplotypes composed by non-intronic regions from 210 *F. asiaticum* isolates. The figure in parenthesis means the number of specific haplotypes.

**FIGURE 4:**
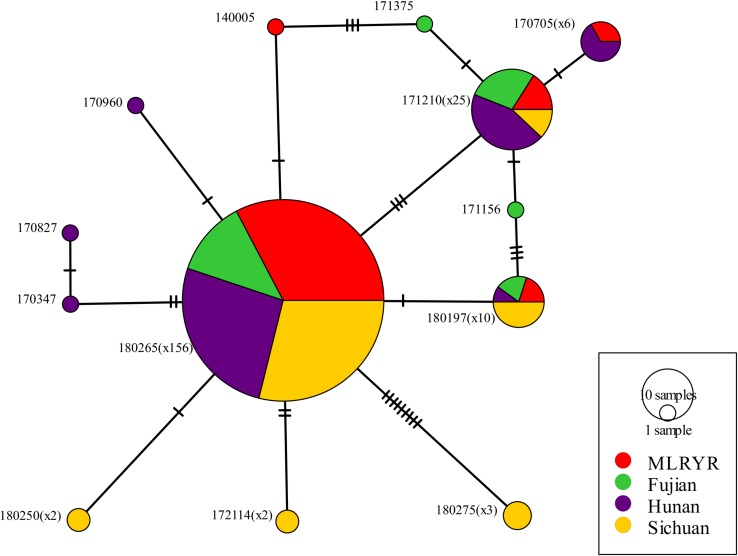
Minimum spanning network inferred from non-intronic regions from 210 *F. asiaticum* isolates. Each perpendicular black line indicates an individual SNP. The number of each haplotype is reflected by size of each circle and by the exact number is given in parentheses following the haplotype codes, except for those haplotypes representing only single isolates.

Parsimony informative sites were extracted from the non-intronic regions of the *F. asiaticum* mitogenome alignment. Excluding gapped sites, this yielded 30 parsimony informative positions out of the 48376 bp-long alignment. Subsequently, the 30 sites were manually curated at which point 8 were removed, because they were due to length variations of neighboring poly tracts, resulting in 22 reliable parsimony informative sites. The curated alignment was used for analysis in PopArt, where 13 haplotypes were detected among the 210 *F. asiaticum* isolates. Composition of haplotypes in four regions was similar with one predominant haplotype found in all regions (180265) (Figure 3). The intronless sequences of *F. asiaticum* are extremely conserved, 156 of 210 strains had the same haplotype (Figure 3). There was no clear grouping between haplotypes and ecological regions nor between haplotypes and hosts (Supplementary Figure S1). However, we found the strains from Sichuan (6/13) and Hunan (7/13) contained more haplotypes than MLRYR (5/13) and Fujian (5/13), and they have more region-specific haplotypes. This revealed that Sichuan and Hunan population were more divergent than the other two. Furthermore, a closed loop was formed by five haplotypes in the network, indicative of recombination in the mitogenomes of *F. asiaticum* population (Figure 4). Detailed analysis of the SNPs involved revealed that the two “side” branches linking the two largest haplotypes differ each by single SNPs in coding sequences that represent synonymous mutations: 140005 and 171375 shared the *cox2*-A234G SNP (position refers to nucleotide position in the coding sequence), while 171156 and the 180197 haplotype group shared the *cob*-C163T SNP. The three additional SNPs involved were the same on all three branches. These three SNPs are adjacent to each other and are located in an intergenic region. Although only five sites are indicative of recombination, they represent 23.8% of the parsimony informative sites used for this analysis. Furthermore, considering the low level of variation observed it is highly unlikely that two SNPs located in coding sequences of highly conserved genes have independent origins or that same three adjacent SNPs mutated the same way in three different backgrounds.

#### Population Analysis of *F. asiaticum* by Intron Pattern

To understand the population structure and estimate the genetic variability of the intron regions of *F. asiaticum*, all 210 strains were genotyped based on their intron composition (Supplementary Table S3): a total of 41 MLGs were identified. The number of MLGs ranged from 16 in Hunan, 17 in MLRYR to 19 in Fujian and Sichuan. In order to eliminate the influence of population size, the normalized multilocus genotypes eMLGs were used to compare the genotypic diversity among the populations. Isolates from Fujian showed the highest genotypic diversity, followed by those from Sichuan, MLRYR and Hunan. The Simpson’s index (lambda) and Nei’s gene diversity index (Hexp) also showed similar pattern compared with the eMLG analysis. In both cases, Fujian and Sichuan showed a higher lambda and Hexp value (Table 1), indicating higher diversity in these populations compared to those from MLRYR and Hunan. All four populations had unique alleles, with the highest frequency of unique alleles found in Sichuan (0.143), followed by Hunan (0.095) and MLRYR (0.071), while particularly in Fujian a low value was observed (0.048) (Table 2). Association indices (rbarD) were used to estimate the recombination within populations. Significant (*P* = 0.0099) linkage disequilibrium with a high rbarD value (0.1758) was observed in Sichuan, indicating limited genetic exchange among strains from this province. Although linkage disequilibrium could be a result of gene flow from other populations, considering that Sichuan shows the highest haplotype diversity and it is a mountainous region limited genetic exchange is the most likely explanation. In the other three regions, the rbarD values were much lower and not significant at the 0.05 level, suggesting genetic exchange among these populations. Pairwise Nei’s genetic distance of the four populations ranged from 0.001 to 0.01 (Table 2). The longest distance was observed between MLRYR and Sichuan, while MLRYR-Hunan and Hunan-Fujian showed the lowest distance.

**TABLE 1:** Summary of population genetic structure of intron regions of 210 *F. asiaticum* mitogenomes in diverse geographical regions.

Pop	N^a^	MLG^b^	eMLG^c^	Private Alleles^d^	lambda^e^	Hexp^f^	rbarD^g^	p.rD^h^
**MLRYR**	60	17	12	0.071	0.836	0.0388	0.0168	0.1980
**Hunan**	60	16	11.9	0.095	0.872	0.0427	0.0104	0.2673
**Fujian**	30	19	19	0.048	0.927	0.0525	−0.0256	0.9505
**Sichuan**	60	19	13.5	0.143	0.983	0.0576	0.1758	0.0099*

*^*a*^N = the number of samples from each region; ^*b*^MLG = number of identified multilocus genotype; ^*c*^eMLG = expected number of MLG at the lowest common sample size; ^*d*^Private Alleles = number of private alleles; ^*e*^lambda = Simpson’s index; ^*f*^Hexp = Nei’s gene diversity index; ^*g*^rbarD = value of the standardized index of association for each population factor; ^*h*^p.rD = *P*-value for rbarD from the number of reshuffles indicated in sample; Asterisks represent significant linkage disequilibrium.*

**TABLE 2:** Matrix of Nei’s Genetic Distance for intron regions of 210 *F. asiaticum* mitogenomes between populations from diverse geographical regions.

	MLRYR	Hunan	Fujian	Sichuan
MLRYR	0.000			
Hunan	0.001	0.000		
Fujian	0.004	0.001	0.000	
Sichuan	0.010	0.006	0.004	0.000

### Interspecies Comparative Mitochondrial Genomes Analyses

#### Variations Between Mitogenomes of *F. asiaticum* and *F. graminearum*

**FIGURE 5:**
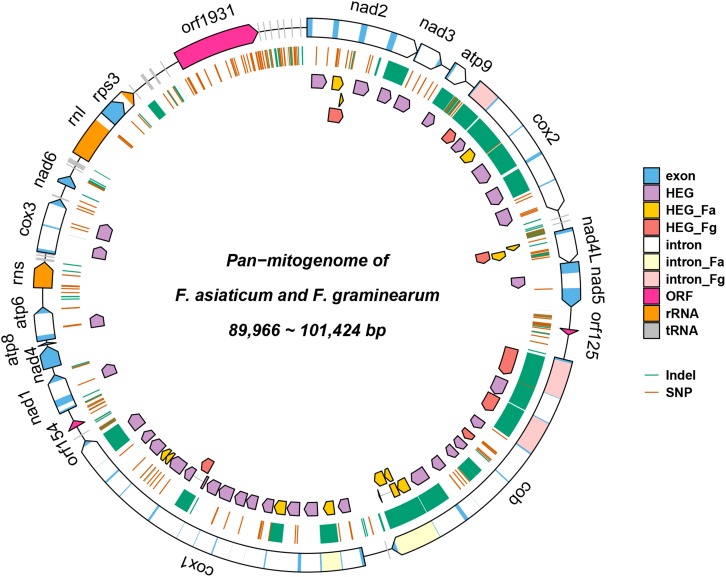
Pan-mitogenome of 210 *F. asiaticum* and 24 *Fusarium graminearum* strains. HEG_Fa and HEG_Fg represent *F. asiaticum* and *F. graminearum* specific HEGs, respectively; intron_Fa and intron_Fg represent *F. asiaticum* and *F. graminearum* specific introns.

**FIGURE 6:**
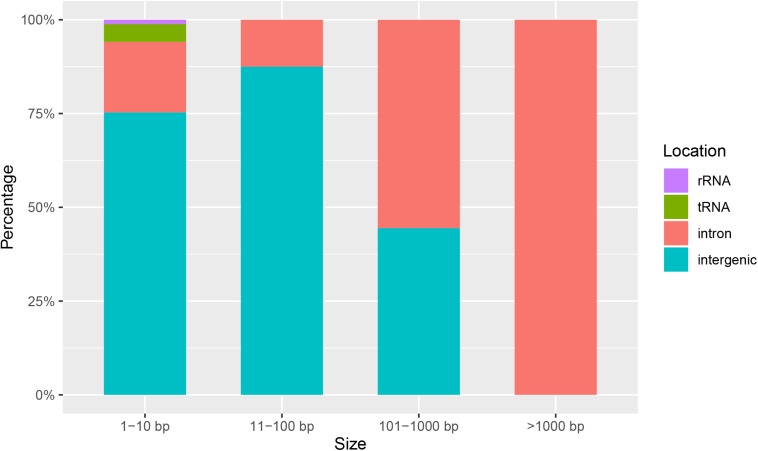
Length distribution of indels in intergenetic, intron, rRNA, and tRNA between different length of intervals in the mitogenome of *F. asiaticum* and *F. graminearum.*

To compare the genetic variation in populations of *F. asiaticum* and *F. graminearum*, we aligned the above 210 *F. asiaticum* and 24 *F. graminearum* mitochondrial sequences and performed a variant calling analysis (Figure 5). A total of 442 variations were identified including 318 SNPs and 124 indels. Most SNPs are located in intergenic (173) and intron (72) regions, others were found in the LV-uORF (34), tRNA (18), rRNA (3), and protein coding sequences (18). Further analysis showed that all the SNPs found in exons were synonymous mutations, but 21 of 34 SNPs in the LV-uORF caused non-synonymous substitutions (Supplementary Table S2). Similarly, 96% of the indels were located in intergenic (82) and intronic (37) regions, while others were in tRNA (4), and rRNA (1), but no indel was found in exon and LV-uORF regions. The length of indels varied from 1 to 2923 bp, but indels with different length were unevenly distributed in different regions (Figure 6). All large indels (>1000 bp) and more than a half of the middle-length indels (100–1000 bp) were located in intron regions. Short indels (<100 bp) were mainly observed in intergenic regions. The length variation of *F. asiaticum* and *F. graminearum* mitogenomes is mainly derived from variation in the intronic regions (Table 3).

**TABLE 3:** The distribution of the length of indels in intergenetic, intron, rRNA and tRNA between different length of intervals in the mitogenome of *F. asiaticum* and *F. graminearum.*

Location	1–10 bp	11–100 bp	101–1000 bp	>1000 bp	Total
Intergenic	64	14	4	0	82
Intron	16	2	5	14	37
rRNA	1	0	0	0	1
tRNA	4	0	0	0	4
Total	85	16	9	14	124
					

#### Intron Analysis

**FIGURE 7:**
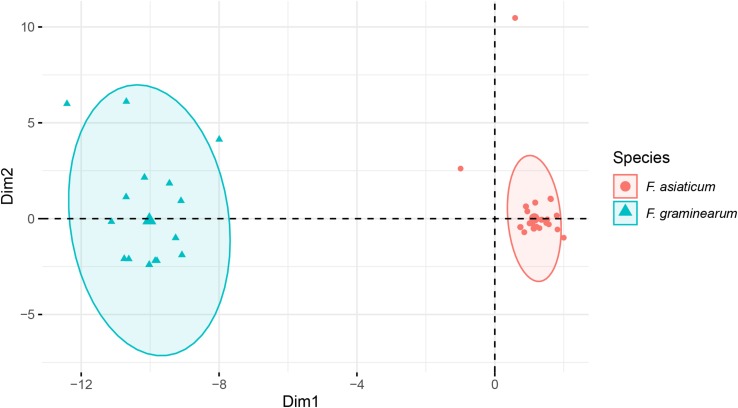
Principal component analysis of intron regions of *F. asiaticum* and *F. graminearum* mitogenomes.

A total of 42 introns was identified in *F. asiaticum* and *F. graminearum* populations, 28 of them were core introns, present in all isolates of the two species, the other 12 showed presence/absence variation. We found two *F. graminearum* specific introns (cox2-i108 and cob-i278) and one *F. asiaticum* specific intron (cob-i823), which were fixed in one species and absent from the other species. Interestingly, some introns showed clear association with the two species. Atp9-i181 and cob-i779 were absent in most *F. asiaticum* strains (209/220), but fixed in the *F. graminearum* population. The size of nad2-i762 was different in the two species, 1475 bp in *F. asiaticum* and 1465 bp in *F. graminearum*. A similar situation was also found in cox2-i228, of which the size in most *F. asiaticum* strains (208/220) was 1146 bp, while a 1159 bp intron was mainly found in *F. graminearum* (16/24) as well as one strain with a 1146 bp intron, one with a 1147 intron bp and six strains with no intron at that location at all. Based on genotypes of intron size, 41 and 15 MLGs were identified in *F. asiaticum* and *F. graminearum* populations respectively. Taking into account the difference of population size, *F. graminearum* showed a higher eMLG value indicating higher genotypic diversity within the population. Simpson’s index, haploid genetic diversity and Nei’s gene diversity index also suggested a higher level of diversity of *F. graminearum* than of the *F. asiaticum* population (Table 4). In a PCA plot, *F. graminearum* isolates distributed in a larger area, whereas most *F. asiaticum* strains were quite close to each other indicating low level of variation. The two species are clearly separated by the PCA analysis (Figure 7). Applying the Mantel test revealed that the geographical and the genetic distances between the populations did not have a linear relationship (*r* = −0.028, *P* = 0.615).

**TABLE 4:** Genotypic diversity of *F. asiaticum* and *F. graminearum* mitogenomes basing intron regions.

Population	N^a^	MLG^b^	eMLG^c^	h^d^	lambda^e^	Hexp^f^
*F. asiaticum*	210	41	12.6	0.051	0.901	0.0511
*F. graminearum*	24	15	15	0.098	0.913	0.1022

*^*a*^N = the number of samples from each region; ^*b*^MLG = number of identified multilocus genotype; ^*c*^eMLG = expected number of MLG at the lowest common sample size; ^*d*^h = Haploid genetic diversity; ^*e*^lambda = Simpson’s index; ^*f*^Hexp = Nei’s gene diversity index.*

#### Phylogenetic Analysis

A maximum likelihood phylogenetic tree was constructed based on the non-intronic (exonic and intergenic regions) alignment of *F. asiaticum*, *F. graminearum* and *F. culmorum*, as outgroup (Supplementary Figure S2). The two species separate into two separate clades with high support.

## Discussion

Fusarium head blight is one of the most devastating diseases in wheat around the world. China has witnessed frequent outbreaks of this disease resulting in serious losses in recent decades, especially in Southern China where *F. asiaticum* was the predominant species on both wheat and barley (Yang et al., 2008, 2018). Our previous study demonstrated that pathogens on rice debris were the primary inoculum of FHB of wheat (Yang et al., 2018). In this study, a large *F. asiaticum* population isolated from wheat kernels or from rice stubble in different ecological regions was sequenced and the mitogenomes of 210 isolates were assembled individually. The size of mitogenomes of *F. asiaticum* was between 89,966 and 98,925 bp, which was similar to the mitogenome size of isolate PH-1 (95,638 bp) of the closely related species *F. graminearum* (Brankovics et al., 2018) and *F. culmorum* (103,844 bp) (Kulik et al., 2016). The sizes and genomic content of mitogenomes among the large number of *F. asiaticum* strains showed a high level of conservation.

In a previous study, we identified 723 variations in 24 *F. graminearum* mitogenomes (Brankovics et al., 2018). In contrast, a significantly lower number of variations (442) was found in a larger set of *F. asiaticum* isolates (*N* = 210) in this study. The genetic variation in the mitochondrial genome is the result of two types of events: (i) small sequence variations (SNPs and short indels) and (ii) intron gain or loss. In our previous study, length variation across *F. graminearum* mitogenomes was found to be largely due to variation of intronic regions (Brankovics et al., 2018). A similar result was observed among the 210 *F. asiaticum* mitogenomes analyzed in this study. In the current research, we found the majority of indels in intergenic regions were very short, whereas all large indels were due to complete or partial gain and loss of introns. The average length of indels in intronic regions was more than seven times longer than those in intergenic regions.

All SNPs detected in the 14 protein-encoding genes of *F. asiaticum* mitogenomes were synonymous. This contrasts to the LV-uORF, a large open reading frame with unknown function that will be discussed in more detail below. High level of conservation was also detected in the analysis of the parsimony informative sites of the non-intronic sequences of *F. asiaticum*, only 13 haplotypes were identified and 74% isolates had the same haplotype. Heterologous recombination in the mitochondrial genome was demonstrated in the asexual species *F. oxysporum* (Brankovics et al., 2017) and also in the sexual reproducing homothallic species *F. graminearum* comparative analyses pinpointed toward recombination events (Brankovics et al., 2017, 2018). The population genomic approach used in the current study allowed a more detailed analyses of the existence of recombination events. The analysis of the non-intronic regions showed a closed network of six haplotypes (Figure 4) demonstrating the occurrence of mitochondrial recombination in *F. asiaticum*.

Several previous studies have reported *F. asiaticum* as the predominant species in the wheat producing areas along the Yangtze River (Zhang et al., 2007; Yang et al., 2018). In a previous study based on variable number of tandem repeats (VNTR) markers, we have shown a high level of genetic diversity and insufficient recombination in the *F. asiaticum* population from the Southwest of China (Sichuan province) which was suggested to be due to geographical barriers observed in this mountainous region and a recent migration of population inferred by unbiased gene flow. In contrast, in middle and lower reaches of Yangtze River Region the pathogen behaved as a random mating population with frequent outcrossing (Zhang et al., 2010, 2012).

Because variation in intronic regions was most prominent, variations in specific introns was used as molecular markers for further population analyses. In agreement with previous population studies performed by VNTR markers (Zhang et al., 2010, 2012), higher genetic diversity and limited genetic exchange were observed in Sichuan, whereas in the MLRYR region a high level of recombination was detected. Hence, we propose that also intron markers can be used for population analyses of *F. asiaticum*. Sichuan province is a mountainous region and the significant linkage disequilibrium there may be due to the geographic isolation of local populations. We did not find strong evidences of a newly introduced population in this study, but the contribution of a recent migration to the high association index should also be considered in respect to previously published data (Zhang et al., 2012). In this study, the Mantel test revealed that no clear geographical patterning of *F. asiaticum* was observed in population differentiation (*P* = 0.615), which is in contrast to our previous study (Zhang et al., 2010). Nevertheless, there are major different factors in two studies, such as different markers, hosts, selection pressure and cropping system, might causing inconsistent consequence.

Two non-wheat growing regions were included in this analysis, this is of interest as we previously reported that the cropping system has a dominant effect on the population structure (Yang et al., 2018), we included two non-wheat growing regions, Fujian and Hunan. Similar as Sichuan, Fujian is also a mountainous region, where higher genetic diversity was observed than the plain region, MLRYR, and the transition region, Hunan. Although a high level of diversity was identified in both Sichuan and Fujian, the type of diversity found was rather distinct. The frequency of specific alleles in both populations varied substantially and recurrent recombination may increase the genetic diversity on a population level even further. Our results revealed that the highest frequency of private alleles contributed most for the high diversity in Sichuan, while the Fujian population showed the lowest value of private alleles, whereas the lowest association index (in Fujian) revealed that diversity was mainly due to more frequent genetic exchange. In addition, linkage equilibrium was also observed in Hunan and MLRYR indicating frequent recombination in the plain and transition regions. Notably, among the three populations with linkage equilibrium, the genetic diversity of rice growing areas in Hunan and Fujian were higher than wheat-rice rotation areas in MLRYR. Wheat has a strong selective effect on different *Fusarium* pathogens in FGSC (Zhang et al., 2016a), and we hypothesize that wheat growing may result in a decrease of the genetic diversity. In contrast, in Hunan and Fujian, *F. asiaticum* can grow saprophytically on rice debris and lack of selective pressure by the host may preserve the high diversity (Yang et al., 2018).

It has been reported that mitochondrial introns can spread through horizontal transfer during sexual and clonal reproduction (Brankovics et al., 2018). The presence/absence frequency of introns could be used to evaluate the spread of these introns. We identified seven introns which showed presence/absence pattern in *F. asiaticum* population. Three of them were at very low frequency, which indicated they have not (yet) dispersed in the population. The absence frequency of the other four introns in each ecological region showed significant relation with population genetic structure. Limited genetic exchange in the Sichuan population may restrict the spread of introns, which resulted in a higher absence frequency of three introns (cox1-i287, cox1-i1287, and cox2-i318) in this region. In the other three regions random mating may have promoted the spread of each of these introns. The specific alleles in the Sichuan population also reflect the low recombination with other regions.

Comparative mitogenomic analyses between *F. asiaticum* and *F. graminearum* revealed more variations than intraspecies of *F. asiaticum*. The LV-uORF is a high variable region in some *Fusarium* species. However, the variations in LV-uORF between *F. asiaticum* and *F. graminearum* were relatively small. Nevertheless, significant more SNPs were found in the LV-uORF region of interspecies than intraspecies comparisons and 20 of 32 SNPs caused non-synonymous substitutions, while no nonsense mutations were obserced. This fuels the debate whether or not the ORF is actually coding for a protein or not. Based on the relatively high number of non-synonymous substitutions it at least seems under a different selection pressure compared to other mitochondrial genes.

As expected, the main variations observed among the *F. asiaticum* mitogenomes were in intronic regions and size differences are primarily related to the presence/absence of introns. All population genetic parameters (eMLG, haploid genetic diversity, Simpson’s index as well as Nei’s gene diversity index) revealed a higher level of diversity in *F. graminearum* compared to *F. asiaticum*. Therefore, we can conclude that the mitogenome of *F. asiaticum* was more conserved than *F. graminearum*. This is also in agreement with the global distribution of the two species. *F. graminearum* is widely distributed all over the world with high genetic diversity. In contrast, *F. asiaticum* seems to be a regional species mainly encountered in East Asia and some confined regions elsewhere, where it coincides with rice production (Suga et al., 2008; Lee et al., 2009; Gale et al., 2011; Zhang et al., 2012; Del Ponte et al., 2015). The smaller geographic distribution and similar environment conditions may lead to a stricter and directional selection, leading to a reduced diversity in the population. However, time and space as well as the scale of sampling can also influence this conclusion. The 24 *F. graminearum* isolates studied by Brankovics et al. (2018) were sampled across ∼100 years, from multiple hosts and originated from all six continents, while all the 210 *F. asiaticum* isolates from the present study originated from Southern China within 4 years. Therefore, additional *F. asiaticum* strains from locations outside China as well as historical collections should be involved in the future to further confirm the conservation of mitogenome of this species. Hybrids between *F. graminearum* and *F. asiaticum* can be generated by *in vitro* crosses (Jurgenson et al., 2002). However, the frequency of occurrence of several species-specific introns as well as PCA analyses revealed a clear species boundary between *F. graminearum* and *F. asiaticum* on a large evolutionary time scale. Moreover, comprehensive surveys were performed in New Zealand (Monds et al., 2005), Japan (Suga et al., 2008), and China (Zhang et al., 2012) but the only natural hybrid encountered so far in the *F. graminearum* species complex was between *F. meridionale* and *F. asiaticum* reported from Nepal (O’Donnell et al., 2000). Our detailed studies on the mitogenome also provide no indication of gene flow between these closely related species.

## Conclusion

Based on the mitochondrial genomic analysis of a large population, we constructed a pan-mitogenome of *F. asiaticum*, which is highly conserved within the species. The length variation was mainly due to presence/absence of intron which harbored homing endonuclease genes. Intron patterns can be used as genetic markers for monitoring population dynamics. Recombination in mitogenomes was identified in *F. asiaticum* as was previously reported for the asexual species *F. oxysporum* and the homothallic species *F. graminearum*. Different levels of genetic diversity and recombination were observed in different ecological regions, which coincided with geographic characteristics and cropping conditions. The interspecies analysis revealed a higher diversity of the global species *F. graminearum* than *F. asiaticum* and clear interspecies differences. The results of this research extend our knowledge on the variability and recombination of mitochondrial genome of *F. asiaticum* and improve our understanding of the evolution and dispersal of this species. It also demonstrates the power of population genomics and the application to track and trace migration using the mitogenome.

## Data Availability Statement

The datasets generated for this study can be found in the GenBank, the accession numbers are listed in Supplementary Table S1.

## Author Contributions

HZ, WC and JF conceived and designed the experiments. MY performed the experiments. MY, HZ, and BB analyzed the data. MY, HZ, BB, TL, CW, and AD wrote the manuscript.

## Conflict of Interest

The authors declare that the research was conducted in the absence of any commercial or financial relationships that could be construed as a potential conflict of interest.

## Footnotes

1 https://github.com/b-brankovics/grabb

2 http://megasun.bch.umontreal.ca/cgi-bin/mfannot/mfannotInterface.pl

3 https://www.ncbi.nlm.nih.gov/orffinder/

4 http://lindenb.github.io/jvarkit/

## Supplementary Material

FIGURE S1 Minimum spanning network inferred from non-intronic regions from 210 *F. asiaticum* isolates. Each black line on lineage indicates one SNP. The number of each haplotype is reflected by area of each circle.

FIGURE S2 A maximum likelihood phylogenetic tree constructed based on the non-intronic (exonic and intergenic regions) alignment of 210 *F. asiaticum*, 24 *F. graminearum*, and one *F. culmorum*.

TABLE S1 Strains information in this study.

TABLE S2 Positions and types of non-synonymous substitutions occurred in the LV-uORF caused by SNPs.

TABLE S3 Intron locations and lengths within standard mitochondrial genes of *Fusarium asiaticum* and *F. graminearum* strains.
