# Supplementary figures and images for: Population Genomic Analysis Reveals a Highly Conserved Mitochondrial Genome in Fusarium asiaticum
Source: Front Microbiol. 2020 May 5;11:839. doi: 10.3389/fmicb.2020.00839 (PMC7214670; doi:10.3389/fmicb.2020.00839)

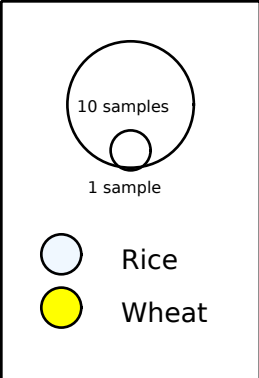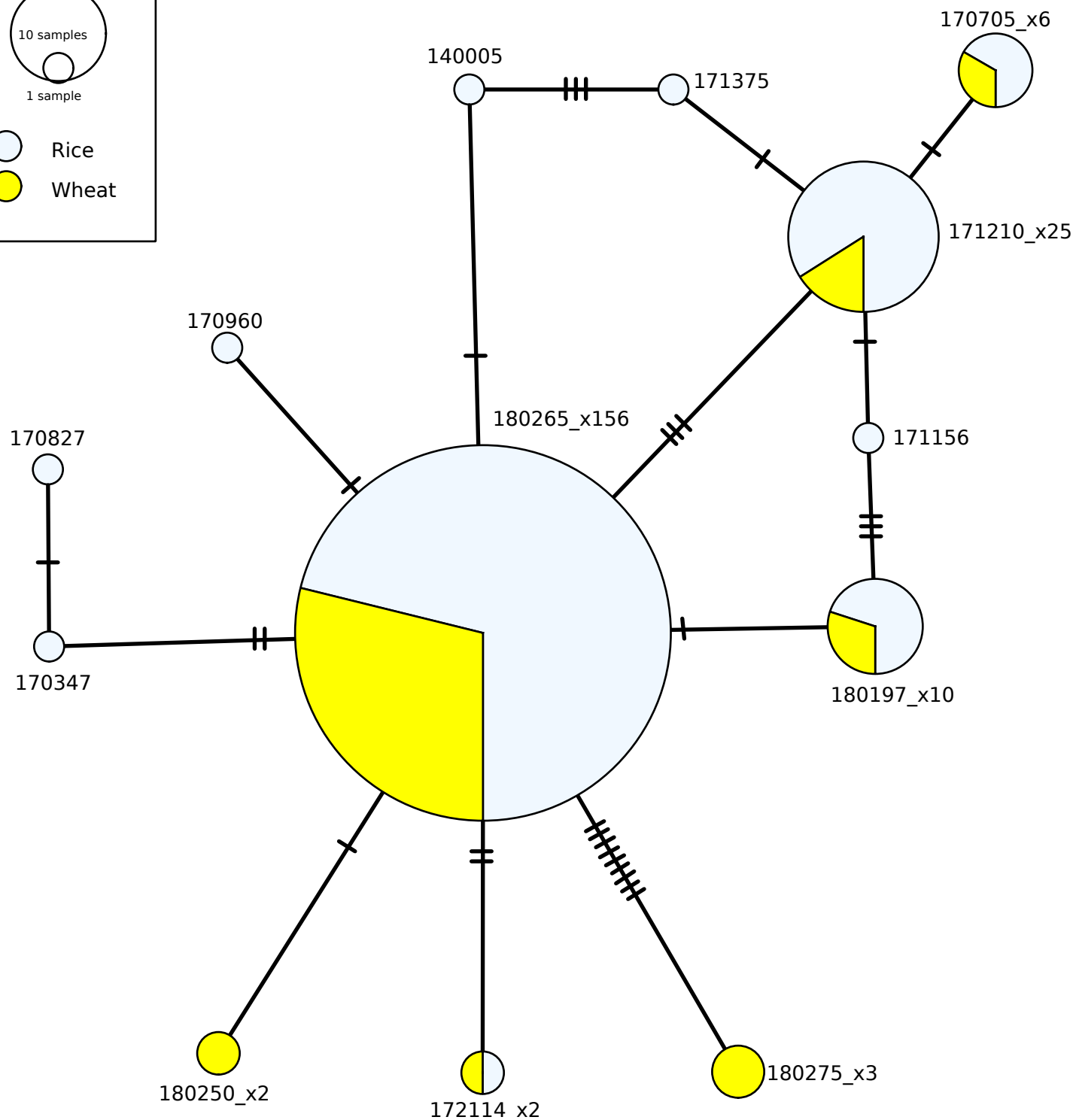

Supplement: FIGURE S1 — Minimum spanning network inferred from non-intronic regions from 210 F. asiaticum isolates. Each black line on lineage indicates one SNP. The number of each haplotype is reflected by area of each circle. [file Image_1.PDF]

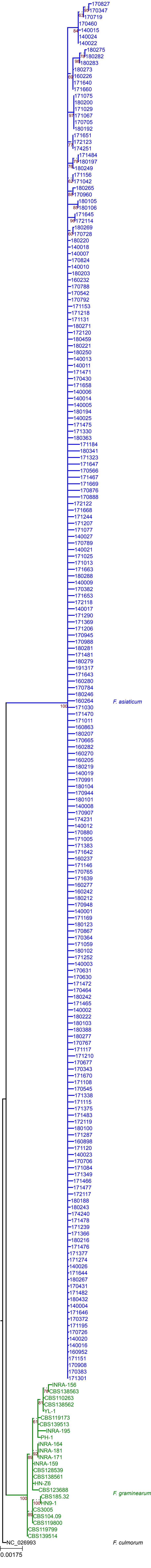

Supplement: FIGURE S2 — A maximum likelihood phylogenetic tree constructed based on the non-intronic (exonic and intergenic regions) alignment of 210 F. asiaticum, 24 F. graminearum, and one F. culmorum. [file Image_2.PDF]
